# Supercritical Carbon Dioxide Impregnation of Gold Nanoparticles Demonstrates a New Route for the Fabrication of Hybrid Silk Materials

**DOI:** 10.3390/insects13010018

**Published:** 2021-12-23

**Authors:** Manish Singh, Estera S. Dey, Sunil Bhand, Cedric Dicko

**Affiliations:** 1 Pure and Applied Biochemistry, Chemistry Deptartment, Lund University, Naturvetarvägen 14, 22362 Lund, Sweden; manish.singh.itbhu06@gmail.com (M.S.); estera.dey1@gmail.com (E.S.D.); 2 Deptartment of Chemistry, Birla Institute of Technology and Science, KK Birla Goa Campus, Pilani 403726, Zuarinagar, Goa, India; sunilbhand@goa.bits-pilani.ac.in

**Keywords:** silk, supercritical carbon dioxide impregnation, nanofiller

## Abstract

**Simple Summary:**

The application of nanotechnology in textiles is limited by the difficulties of loading the fabrics with nanoparticles (NPs) and by their subsequent uncontrolled leakage. More fundamentally, there is a need to answer the question of the “space available” in textile fibers, and generally, other natural polymers for NPs loading. Due to these challenges, there is a risk that uncontrolled leakage of NPs from the textile industry could harm the environment and human health. Here, with a green and straightforward approach, using supercritical carbon dioxide (scCO_2_) as a carrier fluid, we explored the impregnation in four types of Indian textile silks (Mulberry, Eri, Muga, and Tasar) with five standard sizes of gold NPs (5, 20, 50, 100 and 150 nm). The results suggested that all silks could be permanently impregnated with the gold nanoparticles (Au NPs) up to 150 nm. Knowing the available space in silk or other natural polymers can help us understand how and which natural polymers are suitable for use as catalysts, antimicrobial materials, UV-protective agents, and other valuable properties.

**Abstract:**

How many nanoparticles can we load in a fiber? How much will leak? Underlying is the relatively new question of the “space available” in fibers for nanoparticle loading. Here, using supercritical carbon dioxide (scCO_2_) as a carrier fluid, we explored the impregnation in four Indian silks (Mulberry, Eri, Muga, and Tasar) with five standard sizes of gold nanoparticles (5, 20, 50, 100 and 150 nm in diameter). All silks could be permanently impregnated with nanoparticles up to 150 nm in size under scCO_2_ impregnation. Accompanying structural changes indicated that the amorphous silk domains reorganized to accommodate the gold NPs. The mechanism was studied in detail in degummed Mulberry silk fibers (i.e., without the sericin coating) with the 5 nm nanoparticle. The combined effects of concentration, time of impregnation, scCO_2_ pressure, and temperature showed that only a narrow set of conditions allowed for permanent impregnation without deterioration of the properties of the silk fibers.

## 1. Introduction

The primary purpose of impregnating nanoparticles (NPs) in fibers is to increase mechanical strength, improve physical properties, such as electrical conductivity and antistatic behavior, and add functionalities, such as antimicrobial, UV protection, flame retardance, and self-cleaning [1,2,3]. If homogeneously distributed, NPs in polymer matrices can increase the composite toughness and abrasive resistance. There are several ways to impregnate/synthesize nanoparticles, and most importantly, stabilize them on or in fibers. Now, standard methods include plasma treatment [4], in situ synthesis [5], sol-gel synthesis, chemical assembly [6], deposition [7], dip-coating [8,9], radiolysis [10], and sonochemical reduction [11].

The methods above have drawbacks, specifically a decrease in the tensile strength of the fibers; the process demands harsh chemical pretreatment, and leakage of nanoparticles occurs with time [12,13]. The severe chemical pretreatment step and leakage of NPs from fibers lead to serious environmental and health issues [12,13,14].

Within this context, the use of supercritical carbon dioxide (scCO_2_) is an attractive alternative [15,16,17,18,19]. The synthesis/impregnation of NPs on a polymeric substrate under scCO_2_ has several advantages due to some of its unique properties: low toxicity, non-flammable, inexpensive, low surface tension, and no residue in the treated medium after removal [15,20,21,22,23]. Typically, the exposure of natural and synthetic polymers to scCO_2_ results in swelling and enhanced chain mobility of the polymers, which helps to load the additives [24,25,26]. Recent examples and applications using scCO_2_ with dyes [25,27], conductive monomers [28,29], inorganic NPs [30], such as TiO_2_ [31], Ag [32] into fibers have demonstrated the usefulness of the method [33]. However, the leakage, particle stabilization, and impregnation mechanism were unclear.

In 1995, NPs were synthesized on a polymer substrate for the first time using a supercritical fluid [34,35]. Afterward, substantial research led to the incorporation of metal NPs on various inorganic and organic substrates [20,32,35,36,37,38]. Silk has had a limited application with scCO_2_. This is mainly within silk fibers dyeing [25,39], grafting [40,41], and controlled drug delivery application [42]. To the best of our knowledge, no report yet of metal/metal-oxide NPs impregnation in silk fibers using scCO_2_.

Therefore, in the present work, we explored the impregnation in four different types of silks of standardized gold NPs. The choice of gold NPs was motivated by their colloidal stability, monodispersity and low chemical reactivity. For example, a careful evaluation of the plasmon peak of the gold NPs left in solution after impregnation provided a qualitative estimate for their chemical stability. Finally, the choice of size was limited to 150 nm since, at 200 nm, the colloidal stability of the gold NPs was poor.

The combined results demonstrated the usefulness of scCO_2_ for impregnation and determined that the space available in silks was finite if no structural damage was the limiting factor.

## 2. Materials and Methods

Gold NPs of different diameters (5, 20, 50, 100, and 150 nm) were purchased from Sigma-Aldrich (Darmstadt, Germany). The NPs were phosphate stabilized and suspended in 0.1 mM phosphate buffer saline (PBS, Sigma-Aldrich, Darmstadt, Germany), having an optical density of 1 (O.D. = 1). At this O.D. the corresponding concentration were 5 nm (69 µg/mL, 3.5 × 10^−7^ M), 20 nm (53 µg/mL, 2.7 × 10^−7^ M), 50 nm (44 µg/mL, 2.2 × 10^−7^ M), 100 nm (38 µg/mL, 1.97 × 10^−7^ M) and 150 nm (63 µg/mL, 3.2 10^−7^ M). Details are in Table S1. The NPs were used without further treatment. Silk yarns from Mulberry (*Bombyx mori*), Eri (*Samia Cynthia ricini*), Muga (*Antheraea assamensis*), and Tasar (*Antheraea mylitta*) were purchased from Adarsha Traders, Davangere, Karnataka, India. Degummed (soap and sodium carbonate) silk fibers from Mulberry (*Bombyx mori*) were obtained from an online silk supplier (Wild Fibres, http://www.wildfibres.co.uk, accessed on 1 November 2021).

### 2.1. Preparation of Silks Fibers to Be Impregnated with Gold NPs

**Figure 1:**
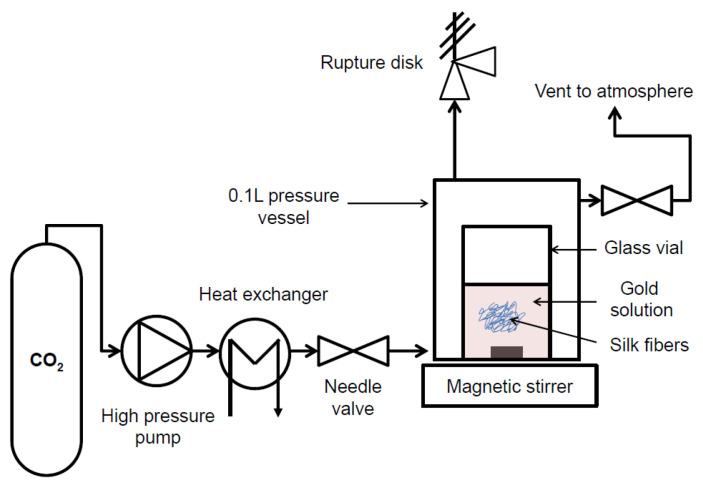
Schematic diagram of supercritical CO_2_ set up. Briefly, when reaching supercritical conditions, the water and gold NPs become a ternary mixture of water/gold NPs/scCO_2_. The new mixture has enhanced diffusion and interfacial properties allowing the gold NPs to be transported in the fibers.

A bundle of fibers was weighed (0.051 ± 0.003 g), gently rinsed with 10 mL of double-distilled water twice and dried at room temperature for 12 h on the lab bench. The dry silk fibers were then immersed in 10 mL of reacting solution (9 mL of water and 1 mL of NPs) in a 20 mL glass vial. We used aluminum foil to cover the glass vial and magnetic bars to mix the solutions (see Figure 1). Total loading would yield the following μg of gold per mg of silk 1.4, 1, 0.9, 0.8 and 1.2 for the 5, 20, 50, 100 and 150 nm, respectively.

### 2.2. Impregnation of Silk Fibers under Supercritical and Atmospheric Conditions

The scCO_2_ impregnation was performed in a modified SFE-100 from Thar Technologies, Inc. (Pittsburg, PA, USA). Figure 1 illustrates the experimental apparatus. Although not intuitive, the CO_2_ in the supercritical state permeates the whole reactor and mixes with the water/g NPs solution. The new mixture (water/gold NPs/scCO_2_) has new diffusion and interfacial properties, transporting the gold NPs onto/into the fibers.

The setup consisted of a steel reaction vessel of 100 mL volume immersed in a thermostated water bath (see temperature details in the results section) with a magnetic stirrer.

The glass vial (20 mL) was inside the reaction vessel. The CO_2_ was pumped in at 11 g per minute to reach the desired pressure. For the impregnation of the four silks, the temperature was kept at 40 °C and the pressure at 200 bars. For the detailed study of the impregnation using the fully degummed Mulberry silk fiber, the final pressure and temperature were adjusted according to the experimental design table (Table 1). The control impregnation happened at atmospheric pressure and at the corresponding temperature and time to match the supercritical conditions. The magnetic agitation speed of impregnation was kept constant at 300 rpm. Each experiment was run in triplicate.

Note that direct attempts with the NPs resulted in the partial coloration of the fibers, thus incomplete and uneven impregnation (data not shown). Henceforth, the impregnation was carried on in the presence of water. The depressurizing effect was not studied, but the return to atmospheric conditions happened as slowly as the instrument allowed (i.e., 20 min). Another result is the known low pH under CO_2_ conditions. Immediately after opening the reaction vessel, we found that the pH of the final solution had a pH of 3. Attempts to maintain the pH at 7 using a 1 M phosphate buffer did not significantly differ. Henceforth, the impregnation happened in water.

### 2.3. Fiber Post-Processing

Washing and fastness test: after the impregnation step, the fibers were recovered for post-processing, namely, washing and fastness treatment successively. The remaining liquid after impregnation was referred to as gold loading. Next, the washing step, the silk fibers, were transferred in a new glass vial and rinsed with 10 mL of water for 1 h. The remaining liquid is referred to as wash leakage. The final step is the fastness test; the silk fibers were transferred to a new glass vial containing 10 mL of water and heated to 50 °C for 1 h. This step is referred to as fastness leakage. The procedure is illustrated in Figure S1 in the Supplementary Information.

The liquid supernatants were measured by UV absorbance, and we calculated the gold loading, wash leakage, fastness leakage, and total efficiency as follows:

(1) $$Gold\;loading\;\left(\%\right)=\left(1-\frac{A_{1}}{A_{0}}\right)*100$$

(2) $$Wash\;leakage\;\left(\%\right)=\frac{A_{2}}{A_{0}-A_{1}}*100$$

(3) $$Fast\;leakage\;\left(\%\right)=\frac{A_{3}}{\left(A_{0}-(A_{1}+A_{2})\right)}*100$$

(4) $$Total\;efficiency\;\left(\%\right)=\frac{A_{0}-\left(A_{1}+A_{2}+A_{3}\right)}{A_{0}}*100$$

where *A*_0_ was the absorbance of gold suspension before impregnation, while *A*_1_, *A*_2_, and *A*_3_ were the absorbance of the liquid supernatant after impregnation, water wash, and fastness treatment, respectively, the efficiencies were statistically compared using a general linear model on the arcsine transformed efficiencies (to avoid truncation) with a Tukey posthoc test for multiple comparisons. The analysis was performed using Minitab (Minitab, Inc., Philadelphia, PA, USA).

### 2.4. Characterization

#### 2.4.1. UV-Visible Absorption Spectroscopy

The UV-Visible (UV-Vis) absorption measurements were performed with a Cary 60 UV-Vis spectrophotometer (Agilent) in the 200–800 nm wavelength range at a scan rate of 600 nm·min^−1^; and a 1 cm plastic cuvette.

#### 2.4.2. Fourier Transform Infrared Spectroscopy-Attenuated Total Reflectance (FTIR-ATR)

The Fourier transform infrared attenuated total reflectance (FTIR-ATR) spectra of silk fibers were measured at different steps: after the impregnation process (scCO_2_ or atmospheric conditions), after washing and after fastness test using a Nicolet iS5 infrared spectrometer with an iD5 ATR accessory with diamond crystal (Thermo Scientific). Each spectrum was background corrected and collected between 550–4000 cm^−1^ (see supporting information Figure S2). Each spectrum was an average of 32 scans at 4 cm^−1^ resolution. The FTIR-ATR spectra were further processed to extract four structural parameters: the crystallinity degree (see Supplementary Materials Figure S3), the tyrosine ratio (see Supplementary Materials Figure S4), Amide I/II ratio and the hydrogen bond index. The silk fibers degree of crystallinity [43] was calculated by comparing the peaks’ intensities at 1263 and 1230 cm^−1^ as follows:

(5) $$Crystallinity\;degree\;\left(\%\right)=\frac{A_{1263}}{A_{1230}+A_{1263}}*100$$

*A*_1263_ and *A*_1230_ are the intensities of the peaks at 1263 and 1230 cm^−1^_,_ respectively.

The tyrosine ratio was calculated by estimating the area under the tyrosine peaks at ±830 and ±850 cm^−1^ and computing the ratio of intensities at 850/830 cm^−1^. The weak features at ±850 and ±830 cm^−1^ make a doublet attributed to the Fermi resonance of the aromatic side chain of the tyrosine residue. The ratio is indicative of the local environment of tyrosine residues within the fibers and, by extension, the local environment of the amorphous regions of the silks [44].

Further, the ratio of the maximum intensities of Amide I (at around 1640 cm^−1^) and II (at about 1510 cm^−1^) peaks was calculated to estimate the total change in secondary structure upon treatment [45,46]. Typically, for silk fibers, the two peaks at ≈1620 cm^−1^ and ≈1510 cm^−1^ are mainly assigned to amide I (C=O and C–N) and amide II (N–H and C–N) β-sheet structures. The ratio will measure any changes due to water hydration and β-sheet structures. Additionally, the Amide I region (1600–1700 cm^−1^) was deconvoluted to extract the secondary structure composition of the silks (see Supplementary Materials Figure S5).

An estimate of hydrogen bond intensity [47] was calculated using the ratio of intensities of N-H vibrations between 3200 cm^−1^ to 3500 cm^−1^. In this region, a careful decomposition of the N-H stretching mode provides some information on the “free” (non-hydrogen bonded ±3400 cm^−1^) and hydrogen-bonded N-H (±3320 cm^−1^). The hydrogen bond index (HBI) was the ratio of bonded to free N-H intensities.

#### 2.4.3. Scanning Electron Microscopy-Degummed Bombyx Mori

Scanning electron microscopy (SEM) images for degummed Bombyx mori were acquired on a JEOL JSM 6700F. The energy-dispersive X-ray spectroscopy (EDXS) analysis was performed using an Oxford X-MAX add-on. The spectra and images were reduced and analyzed using the Aztec software. Before SEM and EDXS, the fibers were carbon-coated.

#### 2.4.4. Photographs

The silk fibers were captured using HP Scanjet G 4050 against a black background.

### 2.5. Factorial Design for Degummed Bombyx Mori Study

To test the effects of temperature, pressure, and time we designed a full factorial table. We measured the following responses: total efficiency and FTIR-ATR results (i.e., Amide I/II ratio, crystallinity, and tyrosine ratio). Table 1 summarizes the factorial table parameters. A total of 17 sample conditions were investigated.

## 3. Results

The four silks chosen are among the most common silks produced in India. Table 2 summarizes their most salient chemical and physical properties [48,49,50].

### 3.1. The Efficiency of the Impregnation Process and Fibers Color Change

**Figure 2:**
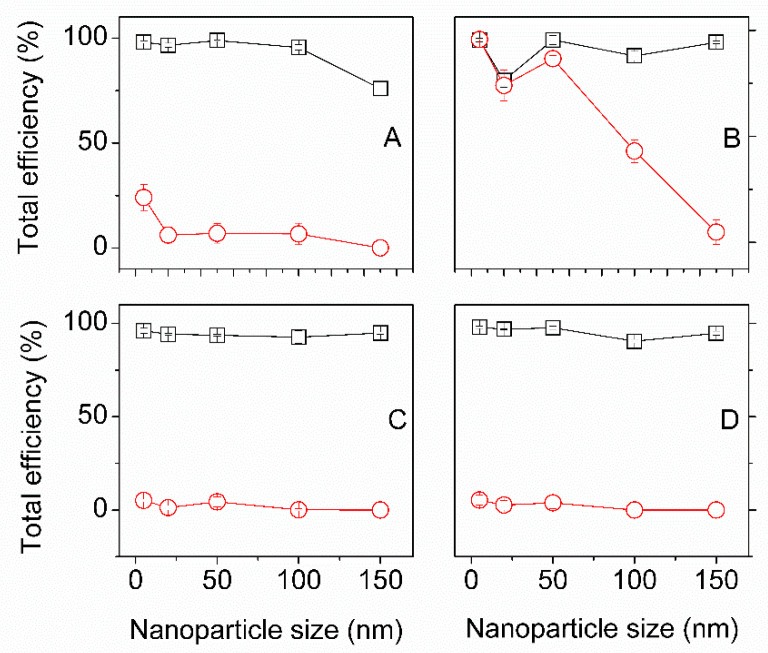
Comparison of total efficiency at supercritical and control impregnation of Indian silks: (**A**) Mulberry (**B**) Eri (**C**) Tasar (**D**) Muga with five different sizes of gold nanoparticle (5, 20, 50, 100 and 150 nm). Black squares (□) are scCO_2_ samples, red circles (O) are control samples. The control and scCO_2_ efficiencies were significantly different (N = 30, *p* < 0, α = 0.05) for Mulberry, Tasar and Muga silks for all gold nanoparticles sizes. For Eri below 100 nm the efficiencies were not significant (N = 30, p_5nm_ = 0.35 p_20nm_ = 0.12 p_50nm_ = 0.09, α = 0.05), above 100 nm the efficiencies were significantly different (N = 30, *p* < 0, α = 0.05).

After correction for wash leakage and fastness leakage (see materials and methods), the total efficiencies are summarized in Figure 2. The total efficiency of gold loading was above 90% for the scCO_2_ treated Mulberry silk fiber with gold NPs size 5, 20, 50, 100 nm, except for 150 nm, where the efficiency dropped to 75.9 ± 3.4%. In the controls, at atmospheric pressure, the total efficiency was constant from 5 to 100 nm at 24.0 ± 6.2%; and dropped to approximately 12% for the 150 nm particle sizes.

For the other three silks, namely: Eri, Tasar, and Muga, the total efficiency of gold loading was above 90% for scCO_2_ impregnated regardless of the gold NPs’ sizes. For those three silks, the scCO_2_ treatment efficiencies were significantly larger (see caption Figure 2). However, Eri silks showed exceptionally high efficiency in the controls at 98.3 ± 0.01, 89.6 ± 2.9, and 94.6 ± 0.7% for 5, 20, and 50 nm size nanoparticles, respectively (not significantly different; see caption Figure 2). Beyond, the total efficiency dropped to 77.2 ± 2.1 and 61.9 ± 2.3% for 100 nm and 150 nm NPs (significantly different; see caption Figure 2).

Tasar and Muga, in the controls, showed a total efficiency of about 10% regardless of the size of the NPs. Increasing concentration, three times more gold at 5 nm, resulted in a decrease in total efficiency for all silks except Eri that stayed constant (see Supplementary Materials Figure S6).

Our observations of the total efficiencies for gold NPs in the four silks studies collectively showed an all-or-nothing effect, which meant that the process parameters have little influence. The four silks behaved identically under scCO_2_ impregnation with no correlation to any of the silk properties shown in Table 2.

The two most remarkable observations were the loss of efficiency for Mulberry after 100 nm. The control sample from Eri silk displayed efficiencies comparable to the scCO_2_ ones and up to 50 nm gold NP. We found, however, no significant properties of Eri that could explain this behavior. A possibility, therefore, was the sericin coating on each of the fibers. There is considerable variability in the sericin from each silk; even though we applied a pre-wash step before scCO_2_ impregnation, we cannot fully control the sericin effect. The sericin is the natural target for dying silk fibers and would likely host the gold nanoparticles. The alternative is to remove the sericin and only use the remaining fibroin brins. The difficulty in sericin removal for Eri, Muga, and Tasar meant that only Mulberry silk fiber was further investigated.

**Figure 3:**
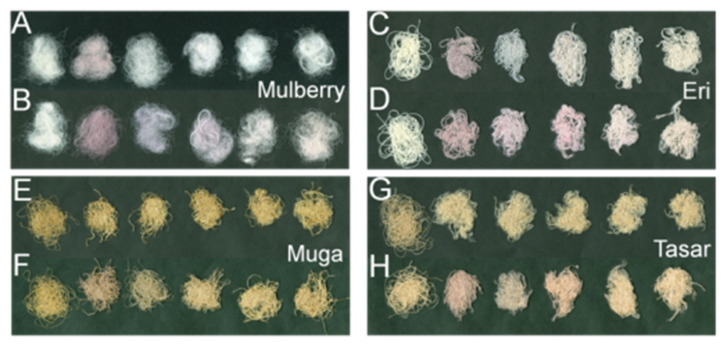
Photographs of the various silks after Gold NPs impregnation. Mulberry: scCO_2_ (**A**), control (**B**); Eri: scCO_2_ (**C**), control (**D**); Muga: scCO_2_ (**E**), control (**F**); and Tasar: scCO_2_ (**G**), control (**H**). The first fiber bundle from the left was the native untreated silk in all photographs. Then the fibers were treated with five different sizes of gold nanoparticles (5, 20, 50, 100, and 150 nm), respectively. The color change correlates with the initial gold solutions’ color.

Figure 3 shows the color changes in the silks from the gold plasmonic effect. Note that the color change for Muga and Tasar silks was not as evident as Mulberry and Eri since the formers are naturally colored.

### 3.2. ScCO_2_ Impregnation Induced Structural Changes

**Figure 4:**
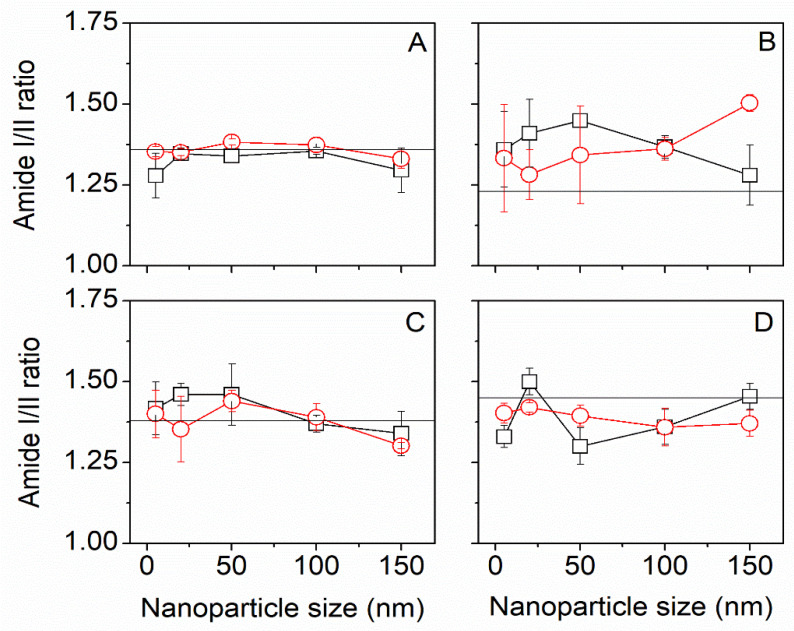
FTIR-ATR change in amide I/ II ratio for Mulberry (**A**), Eri (**B**), Muga (**C**), and Tasar (**D**) silks. Black squares (□) are scCO_2_ samples, red circles (O) are control samples. The horizontal line is the amide I/II ratio in native silk. We found that for scCO_2_ treatment alone, the amide I/II ratio was not significantly different from the native silks.

The evaluation of the impact of the impregnation procedure was principally conducted using FTIR-ATR and XRD. Figure 4 shows the effect of gold NPs size on a global structural parameter: amide I/II ratio. Changes in the amide I/II ratio indicate that a structural change has occurred. For Mulberry silk fiber, we observed no differences between scCO_2_ treatment and control at different gold NPs sizes. Interestingly both traces overlapped with the Amide I /II ratio of the native silk (horizontal line in Figure 4A).

Muga silk behaved similarly. The Tasar silk presented a similar trend to Mulberry and Muga, except that the amide I/II ratio was consistently lower than the native Tasar silk. The Eri silk, on the other hand, the amide I/II ratio showed differences between treatments and departed from the native Eri silk amide I/II ratio.

The Amide I/II ratio changes can be resolved by fitting the Amide I peak with a sum of Gaussian contribution. The position and relative area of the Gaussian peaks were then interpreted in terms of secondary structure content and fraction (Table S2). Four secondary structures were extracted for the four silks, namely, a peak centered at around 1620 cm^−1^ representing intermolecular β-sheets; a peak centered at 1655 cm^−1^ representing a mixture of random coils and α-helices; a peak centered at about 1678 cm^−1^ representing β-turns and a weak peak centered at around 1695 cm^−1^ representing β-sheets structures (see supporting information Figure S5).

In all silks, the β-sheets content appeared constant regardless of the impregnated gold NPs (see Supplementary Materials Figure S7). In Mulberry only, we found an inter-conversion from the random coil/α-helical structures to β-turns with increasing gold NP sizes (see Supplementary Materials Figure S7). Noteworthy is the overlapping signal from water in the Amide I region that may bias the decomposition of secondary structures. For example, scCO_2_ drying followed by washing and high-temperature treatment may affect the water signal contribution in the Amide I differently. Additionally, the contribution of the sericin coating may not be constant throughout the process. These, among others, were the reason for the next section study with the degummed silk.

Besides, we found that the crystallinity index (similar to the β-sheets structure content) did vary marginally from the native silks’ crystallinity, only for Eri, Muga, and Tasar. In Eri, the crystallinity index from the gold impregnated silks was higher than the native Eri fibers but similar to the controls. For Muga and Tasar, we observed the opposite trend, a lower crystallinity (see Supplementary Materials Figure S8). The crystallinity index was independent of the gold NP size in all fibers.

The relative intensity of the tyrosine doublet (Intensity at 850 cm^−1^/Intensity at 830 cm^−1^) was used as a spectral marker of the environment of the hydroxyl groups and the strength of hydrogen bonds involving these groups. The tyrosine residues usually exist in the amorphous regions, containing most amino acids with bulky and polar side chains. An increase in the tyrosine ratio led to the conclusion that the hydrogen bonds involving the tyrosine residues were weak, and consequently, the mobility of the tyrosine residues was higher [44].

For Mulberry, Muga, and Tasar, we found that the tyrosine ratio was constant and closed to the value from the respective native silks (see Supplementary Materials Figure S9). On the other hand, Eri silk showed a constant tyrosine ratio with increasing gold NP size. Still, a systematically lower ratio than native Eri silk, suggesting the amorphous region in Eri was stiffer after the treatment.

**Figure 5:**
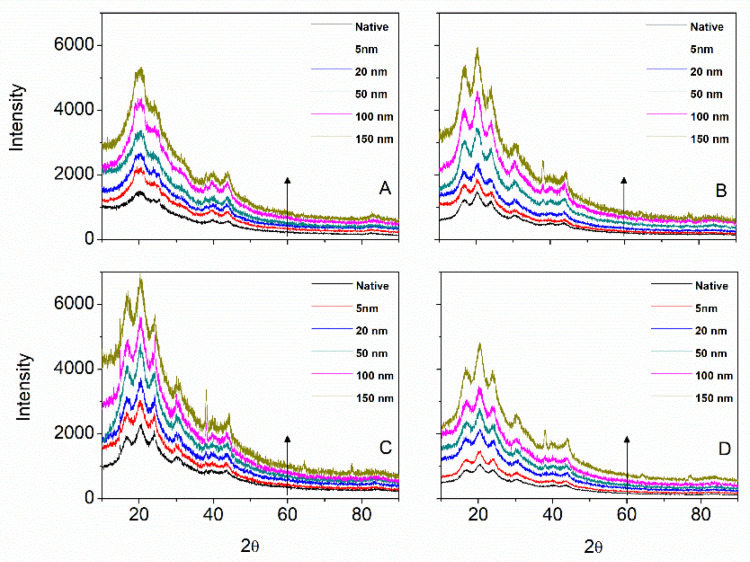
X-ray diffractogram as a function of gold NP size for Mulberry (**A**), Eri (**B**), Muga (**C**), and Tasar (**D**) silks. The arrow indicates an increasing gold NP size. Note at around 37° the (111) reflection for the gold.

The XRD confirmed the gold NPs with an increasing diffraction peak at 38° (Figure 5). The silks diffraction peaks were conserved regardless of the gold NP size used. No further attempt at analyzing the XRD was deemed necessary.

In the next section, we focus only on the Mulberry silk fiber and the 5 nm gold NP to unravel the processes underlying the gold impregnation capacity of silk fibers.

### 3.3. Effect of Degumming on Bombyx Mori and 5 nm Gold NPs

#### 3.3.1. Facile Gold Impregnation in Supercritical Carbon Dioxide at 40 °C and 200 Bars

**Figure 6:**
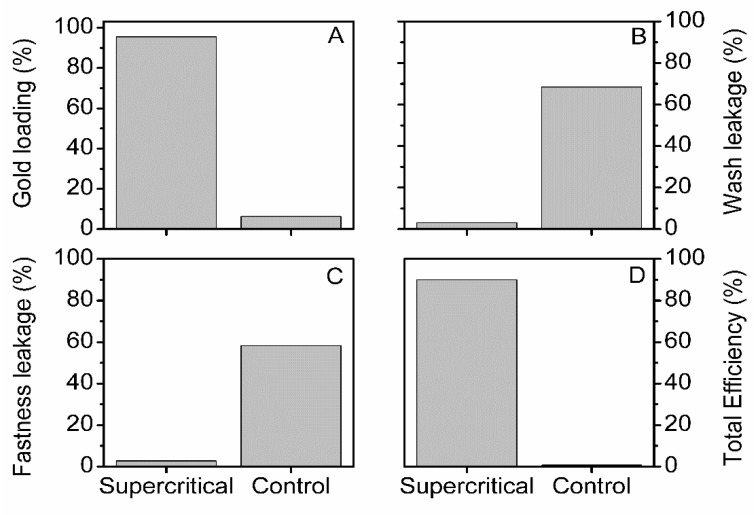
Comparative gold loading (%)—(**A**), wash leakage (%)—(**B**), fastness leakage (%)—(**C**), and total efficiency (%)—(**D**), for supercritical impregnated and control degummed silk fibers. See the materials and methods for details.

The supercritical treatment of silk fibers resulted in a high gold loading (95.5%—Figure 6A), while the control treatment yielded a poor gold loading (6.3%—Figure 6A). The high percentage of wash leakage (68.4%—Figure 6B) and fastness leakage (58.3%—Figure 6C) for the control silk suggested that the gold NPs were weakly attached to the surface of silk fibers.

The scCO_2_ impregnated silk was, on the other hand, in sharp contrast with the control. We found a low percentage of wash leakage (3.0%—Figure 6B) and fastness leakage (2.8%—Figure 6C), thus a small amount of weakly attached gold NPs to silk fibers. The substantial difference in the total efficiency of scCO_2_ and control-treated silk fibers stressed the effectiveness of the former over the latter (Figure 6D).

#### 3.3.2. Effect of Initial Gold NPs Concentration and Time of Impregnation

**Figure 7:**
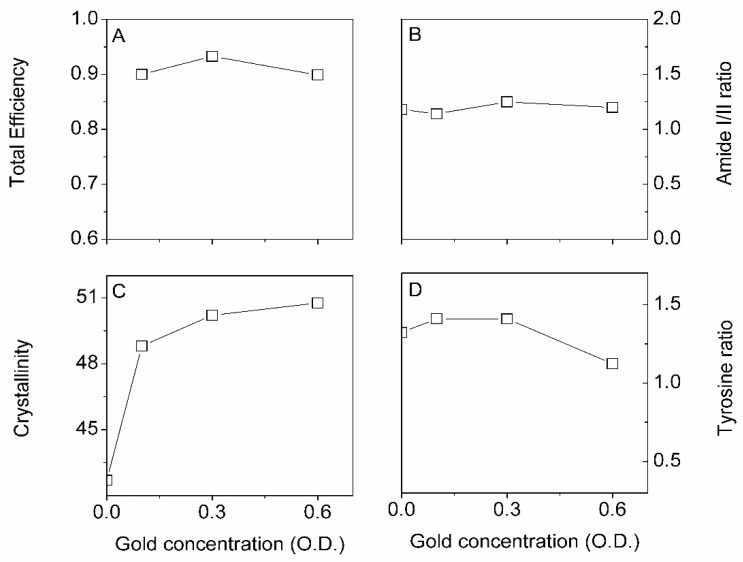
Effect of increasing gold concentration (in optical density O.D.) on total efficiency (**A**), Amide I/II ratio (**B**), Crystallinity (**C**), and Tyrosine ratio (**D**). Interestingly, even though we observed no changes in the amide I/II ratio, we found that the crystallinity and tyrosine ratio were affected. The O.D. concentrations correspond to approximately 6.9, 20.7 and 41.3 µg/mL for the 0.1, 0.3 and 0.6 O.D.

An essential set of parameters is gold NP concentration and time. Figure 7 summarizes our three FTIR-ATR markers and the total impregnation efficiency. Figure 7B–D shows that the first point at t = 0 min represents native degummed silk.

We found that the total efficiency and Amide I/II ratio were independent of the gold NP concentration. On the other hand, the silk crystallinity increased with concentration, whereas the tyrosine ratio decreased. It is important to note that the crystallinity and tyrosine ratio’s positive and negative changes would result in a zero net change in structure, as shown in the amide I/II ratio plot (Figure 7B). The result suggested that some amorphous silk was converted in [-sheet structures (crystallinity)] the typical Silk I to Silk II conversion with increasing gold NP concentration. The existing β-sheets structures were becoming larger through interchain crystallization.

The contribution from the gold NPs to the FTIR-ATR spectra was measured to be at around 1734, 1599, 1448, and 1245 cm^−1^ (data not shown). However, we did not observe significant peaks in those regions, suggesting that silk most likely covered the gold signal.

**Figure 8:**
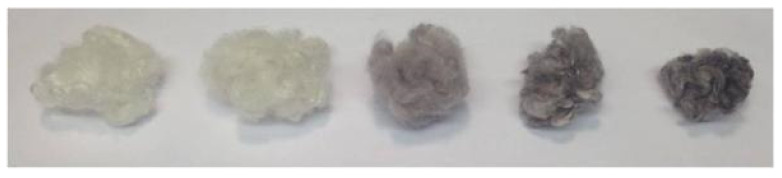
Photograph of impregnated silks, from **left** to **right**: native silk, control impregnation (no scCO_2_), scCO_2_ impregnation 0.1, 0.3, and 0.6 O.D, respectively.

A photograph (Figure 8) illustrates the sharp color change with increasing concentration. 

**Figure 9:**
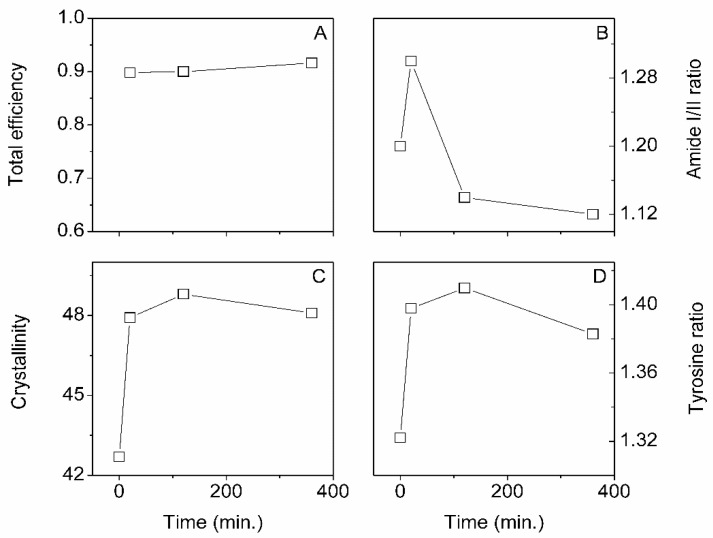
Effect of increasing scCO_2_ impregnation time on total efficiency (**A**), Amide I/II ratio (**B**), Crystallinity (**C**), and Tyrosine ratio (**D**). The gold NP concentration was kept constant at 0.1 O.D.

Figure 9 illustrates the effect of scCO_2_ impregnation time. Similarly to the gold NP concentration, we found that the efficiency was constant at around 90%. Interestingly, the amide I/II ratio decreased sharply with increasing times, whereas the crystallinity appeared steady, and the tyrosine ratio showed a slight decrease with time.

Figure S10 shows the SEM images of a native degummed and scCO_2_ impregnated silk (panel A and C, respectively). EDX spectra of selected features on the silk surface showed no gold for the control and traces of gold for the scCO_2_ (panel B and D, respectively).

**Figure 10:**
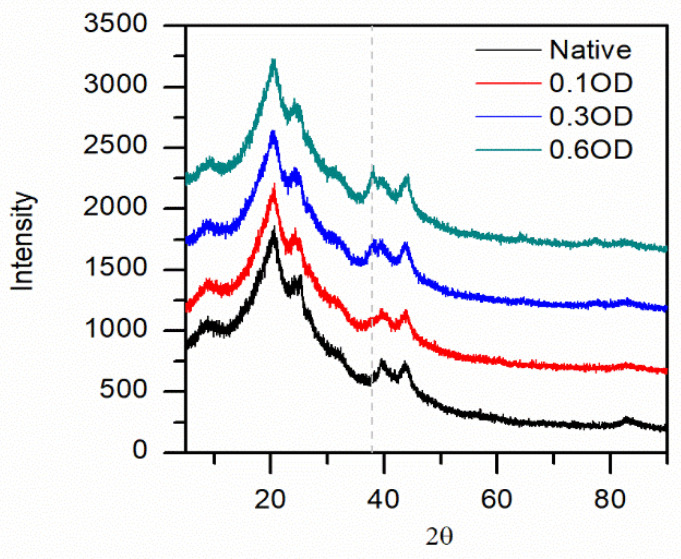
Intensities versus diffraction angle (2θ) as derived by our X-ray diffraction for silk fibers with different amounts of gold. For three times and six times gold addition, the gold peaks were at 2θ = 38°, 65° and 78°. No gold was detected for one-time gold addition. The silk characteristic peaks were at 20°, 25°, 40° and 42°. The grey dotted line indicates the gold reflection at 38°.

Taken together, the low amount of gold NPs on the surface and no gold NPs in the solution, we could suggest that most of the gold NPs were inside the silk, as expected from the efficiencies. A more precise quantification was obtained by XRD (See Figure 10) with the characteristic gold peak at 38° (note that the other peaks at 65° and 78° were barely visible for the highest gold NP concentration used). The distinct silk peaks [48] at 25°, 40°, and 42° appeared unchanged with increasing gold NPs concentrations.

The detailed peak deconvolution of the amide I (see Supplementary Materials Figure S11) showed that the β-sheets structures were constant with gold concentration and impregnation time. We observed, however, a conversion from the random coil/α-helical structures to β-turns with increasing gold concentration and time.

Throughout the analysis so far, we found that changes happened in the amorphous region of the silk. A complementary analysis of the interactions within the silk structures was the hydrogen bond index (HBI) from N-H vibration (ratio of intensities at 3320 to 3400 cm^−1^). Alternatively, one could use the carbonyl signal C=O between 1600 to 1700 cm^−1^. The presence of other strongly overlapping bands in the C=O region precluded a correct estimation of the HBI.

**Figure 11:**
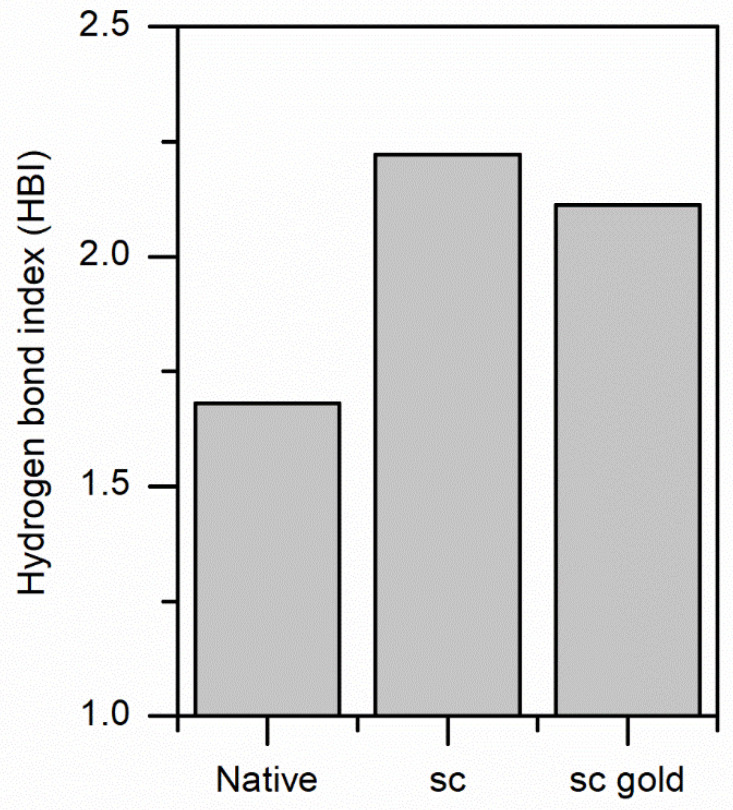
Change in Hydrogen bond index for N-H vibration for native silk, scCO_2_ only treated silk, and scCO_2_ plus gold NPs impregnated silk.

The results for the HBI of the three samples (Figure 11) suggested that the hydrogen bond index increased relative to the native silk but was not different from the two scCO_2_ treated silks. Overall, the structural effects observed were predominantly coming from scCO_2_ treatment.

To help understand further the effect of the scCO_2_ process parameters (temperature, time, pressure, and mixing), we used a factorial design.

### 3.4. Factorial Design—Relationships between Process Parameters and Multiple Responses

In the factorial design table (see Table 1), we combined the various factors of interest at different levels. We explored our four typical responses: total efficiency, crystallinity, Amide I/II ratio, and tyrosine ratio.

We found for the total efficiency that only pressure and time contributed significantly (significance level α = 0.1) to explain the total efficiency variance. We also noted that both pressure and time contributed equally. For the Amide I/II ratio, we found that pressure and time were significant, except that pressure had a more substantial effect than time. For the crystallinity, only the temperature contributed significantly to the effect. Eventually, only the time had a more significant effect on the tyrosine ratio, and the pressure was marginally significant (see Supplementary Materials Figure S12).

One advantage of the factorial design is that one can seek the optimal conditions for a set of parameters. Optimally, we wished to maximize efficiency while maintaining the crystallinity at the lowest level possible. We found an optimal set of conditions: 35 °C for temperature, 250 bar for pressure, 1 h for the time, and 300 rpm for the mixing speed.

## 4. Discussion

### Mechanism of Impregnation and Gold Nanoparticles Location

The permeation of scCO_2_ into a polymer causes it to swell. Aided by its zero-surface tension, the addition of scCO_2_ into the polymer phase gives the chains higher mobility. The CO_2_ molecules act as lubricants, which reduce chain-chain interactions by increasing the polymer’s inter-chain distance and free volume [53,54,55,56], also known as plasticization. The physical properties of the polymer are changed dramatically, including the depression of the glass transition temperature (Tg), the lowering of interfacial tension and a reduction of the viscosity of the polymer melt. ScCO_2_ may increase the crystallinity of the polymers because the polymer chains are freer to align themselves with a more favorable order [57]. The above phenomena describe well the impregnation of soluble molecules into polymers. In the case of NPs, however, little is known.

Nevertheless, the studied silks could host gold NPs with sizes up to 150 nm using SCO_2_. However, it is unclear as to the location specificity of the gold NPs with size. For example, would the smaller NPs be preferentially located more in-depth, and, as the size increases, the NPs would be nearer the silk surface.

**Figure 12:**
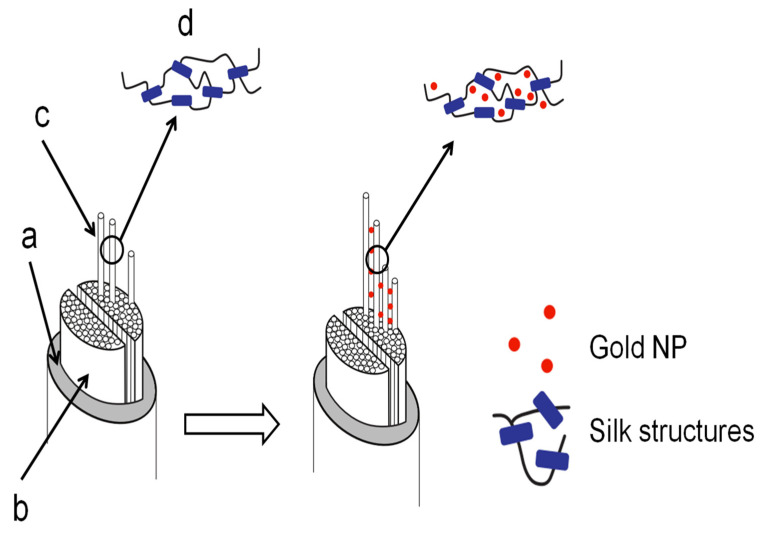
Gold NPs impregnation in silk. The typical silk fiber consists of (**a**) sericin coating, (**b**) fibroin brins, (**c**) fibrils, and (**d**) the secondary structure (idealized here the β-sheet as blue rectangles and the α-helices, random coils and turns as curvy black lines). After impregnation, the gold NPs were found in the inter-fibrillar spaces.

The details of the degummed Mulberry silk fiber impregnation mechanism suggested that the gold NPs were limited to the silk inter-fibrillar space and, more specifically, around the amorphous regions. Figure 12 illustrates our findings.

Further research on ternary systems comprising “CO_2_ + Nonsoluble NPs + fibers (solid substrates)” is required for a detailed understanding of mass transfer and diffusion in the substrate and of influences on the properties of the bulk material as crystallinity, morphology, anisotropy, and reactivity [58].

Collectively our results lead to the hypothesis that, under the supercritical conditions used in our experiment, the transport of the NPs would happen due to a gradient in surface tension. Park et al. [59] found, for example, that polystyrene (a polymer close to silks) surface tension decreases with CO_2_ increased solubility in the polymer (i.e., higher pressures and higher temperatures). They also found that the polymer surface tension was independent of the polymer conformational entropy; in other words, its internal organization.

One could envision a transport phenomenon akin to the Marangoni flow [60]. The nature of the gradient remains unclear.

## 5. Conclusions

In conclusion, we demonstrated the permanent loading of gold NPs in four types of silk (Bombyx, Eri, Tasar, and Muga) using green and scalable technology like scCO_2_. This work addressed the existing problem of uncontrolled leakage of the loaded particles from fibers, which is a significant concern for the environment and a hurdle in nanotechnology applications for synthetic and natural fibers’ functionalization.

Further, we reported space availability in silk fibers by scCO_2_ assisted impregnation at low temperatures. The four silks (Mulberry, Eri, Muga, and Tasar) displayed a remarkable capacity for the size of gold NPs (up to 150 nm). The detailed study of the impregnation mechanism in degummed mulberry silk fiber suggested a narrow window of process parameters with no detrimental effect on the fiber. The mechanism of impregnation of NPs into a solid fiber substrate is yet to be resolved; we hypothesized that the transport of the NPs was possible because of a surface tension gradient at the liquid-solid interface.

This developed approach is scalable, environmentally friendly. The results could help predict the application of natural fiber loaded with NPs as catalysts, self-cleaning, antimicrobial materials, UV-protective agents, and other valuable properties.

## Figures and Tables

**Table 1:** Factorial design parameters.

Factors	Levels	Range
A: Temperature (°C)	2	35, 50 and center point at 42.5
B: Pressure (bars)	2	100, 250 and center point at 175
C: Time (hours)	2	1, 3 and center point at 2
D: Mixing (rpm)	2	0 and 300

**Table 2:** Silk properties.

	Mulberry	Eri	Muga	Tasar
Average density (g/cm^3^) ^a^	1.357	1.288	1.34	1.323
Average moisture regain (%) ^a^	7.04	8.03	7.61	8.52
Sericin content (%) ^b^	10.4–24.4	6.5–10.1	8.6–12.7	8.2–14.4
Acid dye exhaustion (%) ^c^	89.82	58.38	57.02	59.10
Disperse dye exhaustion (%) ^d^	23.67	13.55	12.59	16.20
Elongation (%)	13.5	20.8	22.3	26.5
Tenacity (g/d) ^e^	3.75	3.7	4.35	4.5
Initial modulus (g/d)	95	89	81	84
X-ray crystallinity (%)	38.2	32.6	35.0	35.2
Glass transition (°C) ^f^	200–220	220–235	215–235	235–250
Basic/Acidic ratio ^g^	0.65	1.3	1.24	0.97
Hydrophilic/hydrophobic ratio ^g^	0.28	0.35	0.38	0.44
Bulky/non bulky side groups ratio ^g^	0.17	0.24	0.28	0.33
Glycine/Alanine ratio ^g^	1.58	0.8	0.82	0.81

^a^ average of outer, middle and inner silk cocoons layers. ^b^ low and high sericin content in fibers (from reference [50]). ^c^ Texacid fast red A (acid dye). ^d^ Foron scarlet S-3GFL (disperse dye). ^e^ g/d: gram per denier. ^f^ from references [51,52]. ^g^ from reference [50].

## Data Availability

The data presented in this study are available in the manuscript.

## Supplementary Materials

The following are available online at https://www.mdpi.com/article/10.3390/insects13010018/s1, Figure S1: wash procedure and leakage calculation. Figure S2: Typical FTIR-ATR spectra of Mulberry, eri, muga, and tasar silks., Figure S3: Crystallinity index. The spectrum is truncated between 1180 and 1300 cm^−1^, and a linear baseline is subtracted. The crystallinity index is computed using the intensities of the peaks at 1263 and 1230 cm^−1^ (red stars). Figure S4: Tyrosine ratio estimate. The spectra are truncated between 790 and 870 cm^−1^, and a linear baseline is subtracted. The ratio is computed from the peak maxima at around 830 and 850 cm^−1^ (red stars in the figure). Figure S5: Secondary structure decomposition and analysis. Typical peak deconvolution of the Amide I and Amide II (top panel) and associated residuals (bottom panel). Briefly, a series of Gaussian peaks are fitted simultaneously to the Amide I and II. The initial position of the peaks is determined by secondary derivative analysis [37]. The final number of peaks is determined using an F-test to compare different models. The Amide I/II ratio is computed directly from the two main peaks in the upper panel. Figure S6: Comparison of total efficiency of 5 nm gold NP impregnation at 0.1 and 0.3 O.D. Figure S7: FTIR-ATR changes in secondary structure content as a function of Au NP size for Mulberry (A), Eri (B), Muga (C), and Tasar (D) silks. Black squares (□) are inter β-sheets, red circles (O) are β-turns, blue triangles (∆) are α-helices and random coils, inverted green triangles (∇) are intra β-sheets. Figure S8: FTIR-ATR change in crystallinity for Mulberry (A), Eri (B), Muga (C), and Tasar (D) silks. Black squares (□) are scCO_2_ samples, red circles (O) are control samples. The horizontal line is the crystallinity in native silk. We found that for scCO_2_ treatment alone, the crystallinity was not significantly different from the native silks. Figure S9: FTIR-ATR changes in tyrosine ratio for Mulberry (A), Eri (B), Muga (C), and Tasar (D) silks. Black squares (□) are scCO_2_ samples, red circles (O) are control samples. The horizontal line is the tyrosine ratio in native silk. We found that for scCO_2_ treatment alone, the tyrosine ratio was not significantly different from the native silks. Figure S10: SEM images of native (A, scale bar 5 µ) and impregnated (C, scale bar 10 µ) silk fibers. The white squares represent the areas where the EDX spectra were collected. EDX spectra of native (B) and impregnated (C) silk fibers. The spectra showed only weak gold signals at 2.5, 7, 9.5, 10.5, 11.5, and 13.5 keV (D). Figure S11: FTIR-ATR changes in secondary structure content as a function of Au NP concentration. Black squares (□) are inter β-sheets, red circles (O) are β-turns, blue triangles (∆) are α-helices and random coils, inverted green triangles (∇) are intra β-sheets. Figure S12: Pareto charts of the standardized effect from the full factorial analysis. The red dotted line is the standardized value above which a factor or combination of factors is considered significant (α level = 0.1). Table S1: Gold nanoparticles concentration and mass. Table S2. Consensus assignment for silk secondary structures determination by FTIR.
