# Supplementary material for: Supercritical Carbon Dioxide Impregnation of Gold Nanoparticles Demonstrates a New Route for the Fabrication of Hybrid Silk Materials
Source: Insects. 2021 Dec 23;13(1):18. doi: 10.3390/insects13010018 (PMC8777700; doi:10.3390/insects13010018)
Supplement: Supplementary file 1 [file insects-13-00018-s001.zip › insects-1477143-supplementary.pdf]

Gold Nanoparticles Uptake and Reduced Leakage Dependent on Access to Size-Dependent Internal Volumes in Silk Fibers

Manish Singh [1], Estera S. Dey [1], Sunil Bhand [2] and Cedric Dicko [1,*]

[1] Pure and Applied Biochemistry, Chemistry Deptartment, Lund University, Naturvetarvägen 14, 22362 Lund, Sweden; manish.singh.itbhu06@gmail.com (M.S.); estera.dey1@gmail.com (E.S.D.)
[2] Deptartment of Chemistry, Birla Institute of Technology and Science, KK Birla Goa Campus, Pilani NH 17B, Zuarinagar, Goa, India; sunilbhand@goa.bits-pilani.ac.in
* Correspondence: cedric.dicko@tbiokem.lth.se

Supplementary information

Particles sizes and concentration

Table S1. Gold nanoparticle concentration and mass.

Diameter	OD	Concentration (Particles/mL)	Mass of Gold (µg/mL)	Molarity (M)
5	1	4.92–6.01 10^{13}	68.9	3.50E-07
20	1	5.89–7.19 10^{11}	52.9	2.69E-07
50	1	3.16–3.86 10^{10}	44.2	2.25E-07
100	1	3.45–4.22 10^{9}	38.7	1.97E-07
150	1	3.20–4 10^{9}	63.0	3.20E-07

Leakage procedure

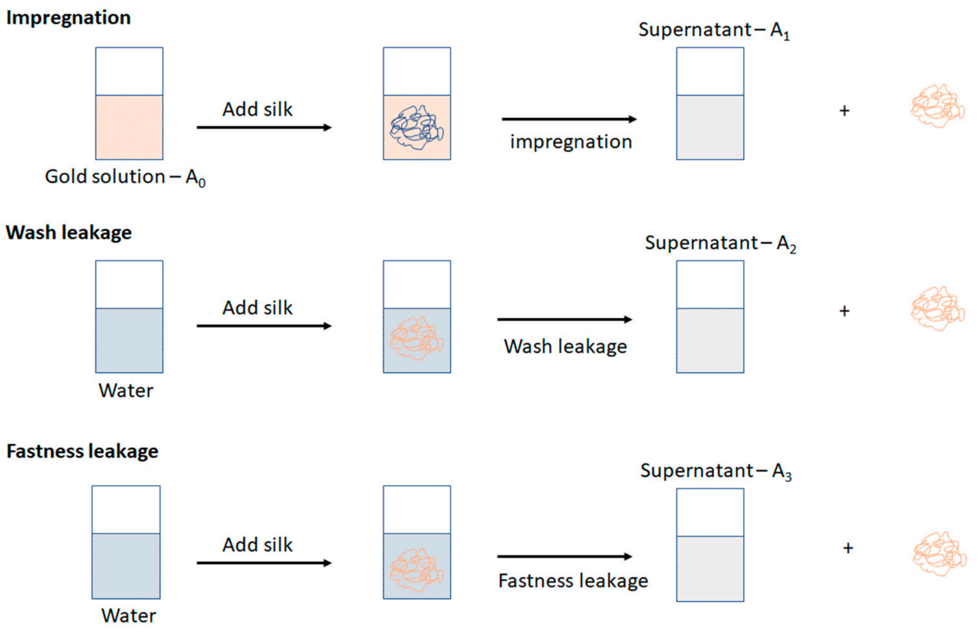

Figure S1. Illustration of the fiber post-processing. First step, the impregnation of a gold nanoparticle solution (absorbance A_0). After SCO_2 impregnation, the leftover gold absorbance is measured as A_1: the gold loaded. The second step, a simple wash in water. The supernatant after the wash as a gold absorbance of A_2: the wash leakage. The third step, the fastness wash, at 50° in water for 1 hour. The supernatant after the fastness wash as a gold absorbance of A_3: the fastness leakage.

FTIR-ATR

Typical silk FTIR-ATR spectra and regions of interest

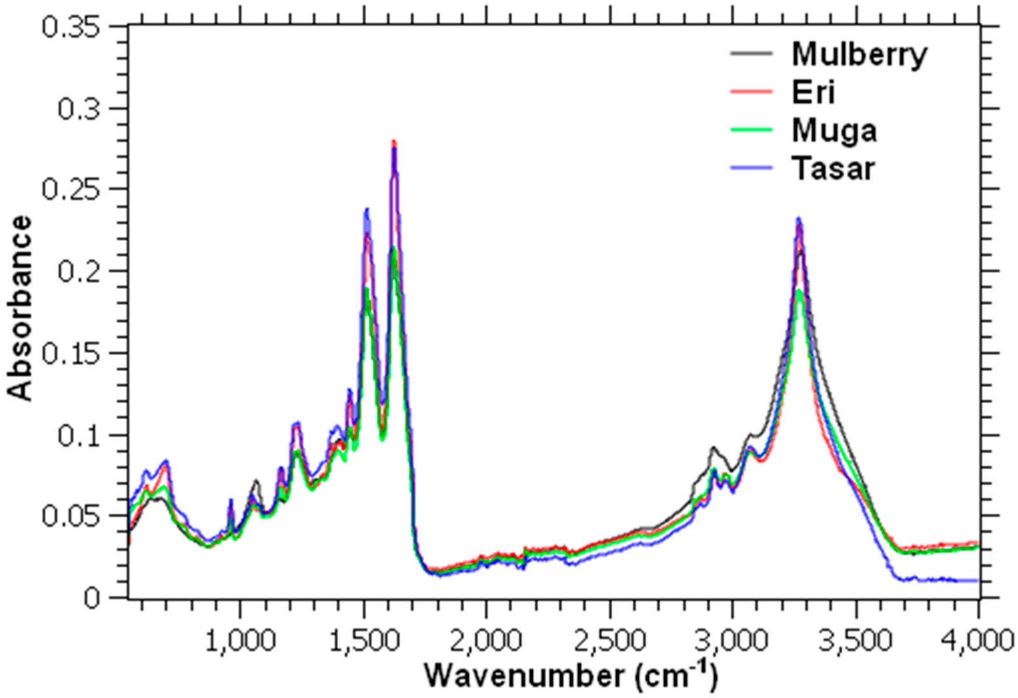

(A)

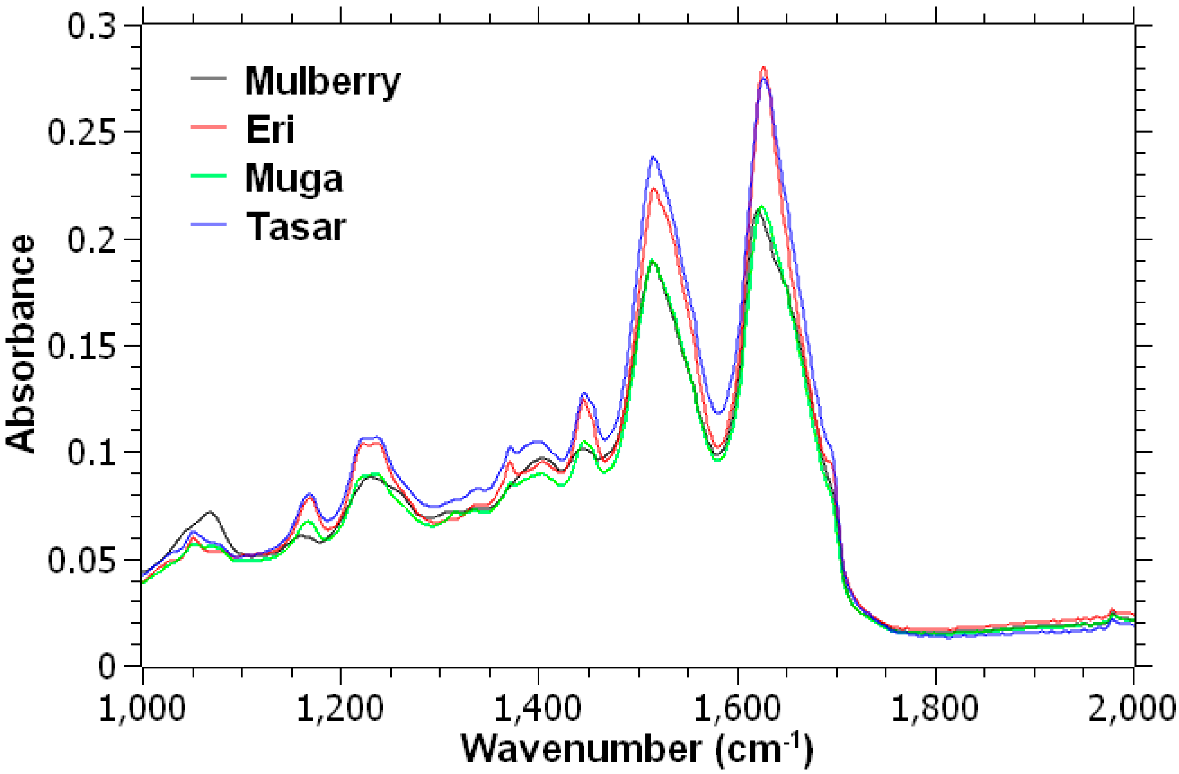

(B)

Figure S2. (**A**). Typical FTIR-ATR spectra of mulberry, eri, muga, and tasar silks. (**B**). Typical FTIR-ATR spectra of mulberry, eri, muga, and tasar silks.

Crystallinity index

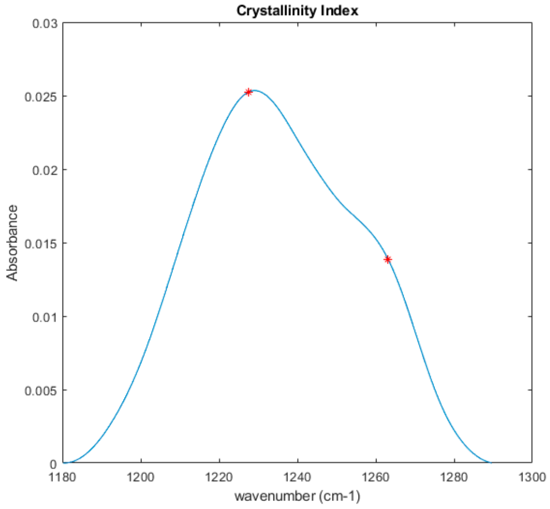

Figure S3. Crystallinity index. The spectrum is truncated between 1180 and 1300 cm^{-1}, and a linear baseline is subtracted. The crystallinity index is computed using the intensities of the peaks at 1263 and 1230 cm^{-1} (red stars).

Tyrosine ratio

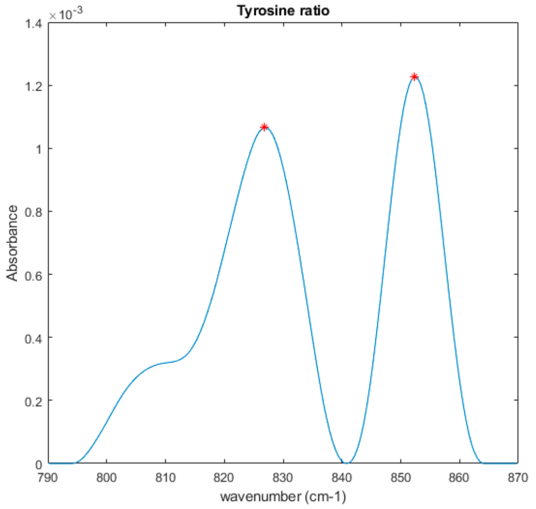

Figure S4. Tyrosine ratio estimate. The spectra are truncated between 790 and 870 cm^{-1}, and a linear baseline is subtracted. The ratio is computed from the peak maxima at around 830 ad 850 cm^{-1} (red stars in the figure).

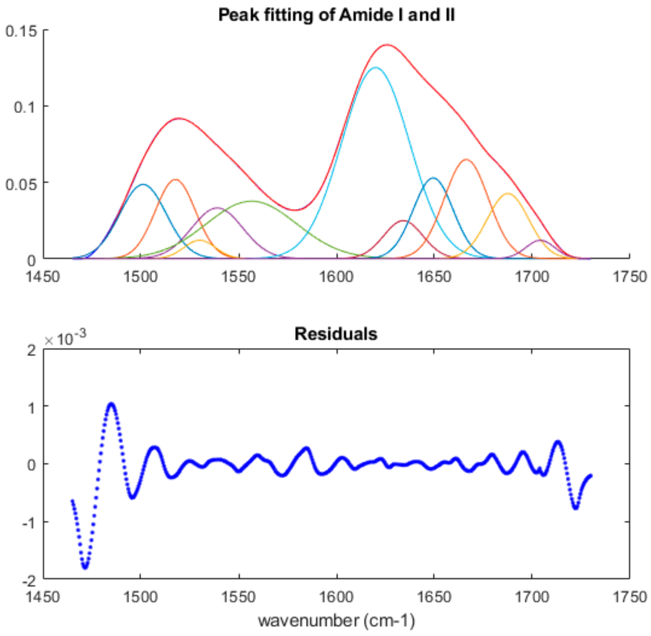

Figure S5. Secondary structure decomposition and analysis**.** Typical peak deconvolution of the Amide I and Amide II (top panel), and associated residuals (bottom panel). Briefly, a series of Gaussian peaks are fitted simultaneously to the Amide I and II. The initial position of the peaks is determined by secondary derivative analysis [37]. The final number of peaks is determined using an F-test to compare different models. The Amide I/II ratio is computed directly from the intensities of the two main peaks in the upper panel.

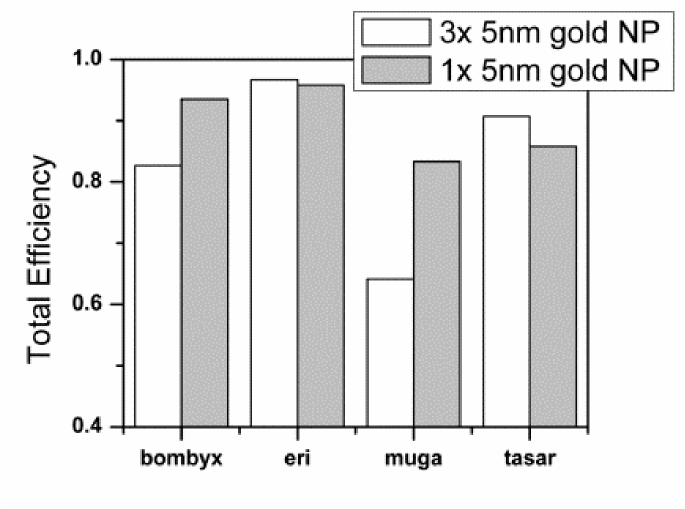

Figure S6. Comparison of total efficiency of impregnation of 5 nm gold NP at 0.1 and 0.3 O.D.

To determine the secondary structure content, we used a consensus assignment of vibrational bands in silks. Table S1 summarizes the current consensus.

Table S2. Consensus assignment for silk secondary structures determination by FTIR.

Wavenumber Range (cm^{-1}).	Assignment
1605–1615	(Tyr) side chains/aggregated strands
1616–1621	aggregate beta-strand/ beta-sheets (weak) [a]
1622–1627	beta-sheets (strong) [a]
1628–1637	beta-sheets (strong) [b]
1638–1646	random coils/extended chains
1647–1655	random coils
1656–1662	alpha-helices
1663–1670	turns
1671–1685	turns
1686–1696	turns
1697–1703	beta-sheets (weak) [a]

[a] Intermolecular beta-sheets
[b] Intramolecular beta-sheets

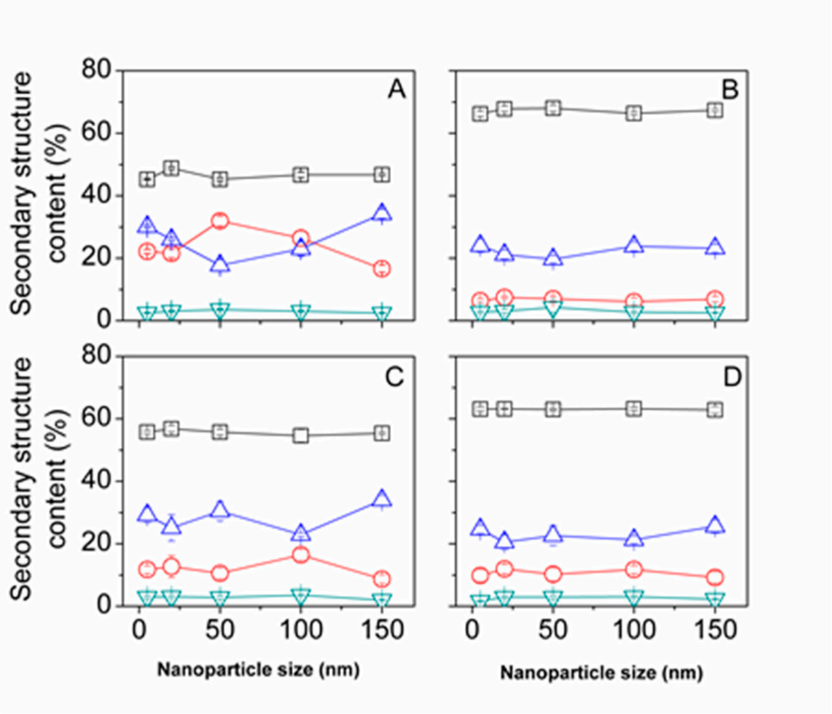

Figure S7. FTIR-ATR changes in secondary structure content as a function of Au NP size for Mulberry (A), Eri (B), Muga (C), and Tasar (D) silks. Black squares (□) are inter β-sheets, red circles (O) are β-turns, blue triangles (Δ) are α-helices and random coils, inverted green triangles (∇) are intra β-sheets.

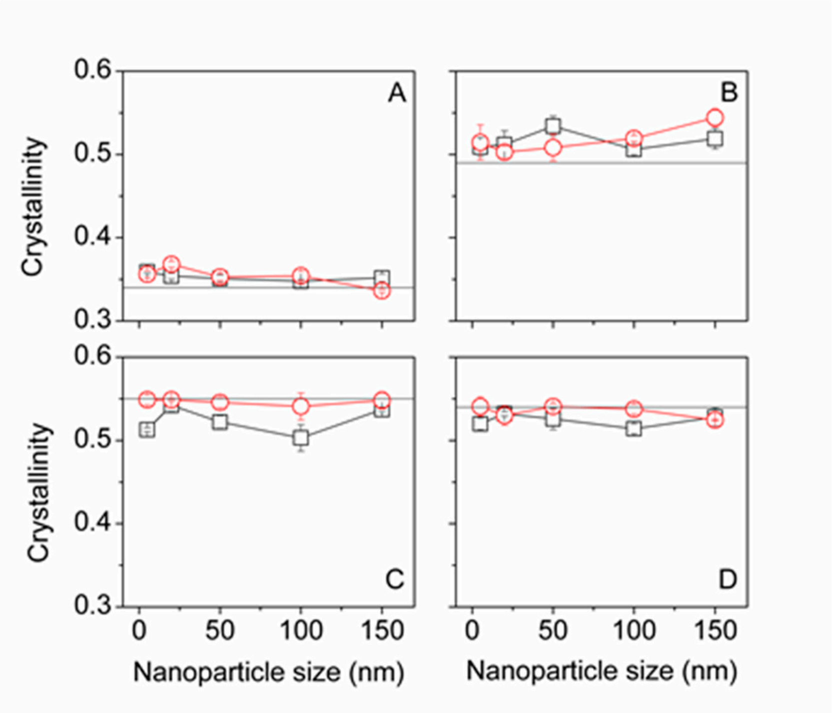

Figure S8. FTIR-ATR change in crystallinity for Mulberry (A), Eri (B), Muga (C), and Tasar (D) silks. Black squares (□) are $scCO_2$ samples, red circles (O) are control samples. The horizontal line is the crystallinity in native silk. We found that for $scCO_2$ treatment alone, the crystallinity was not significantly different from the native silks.

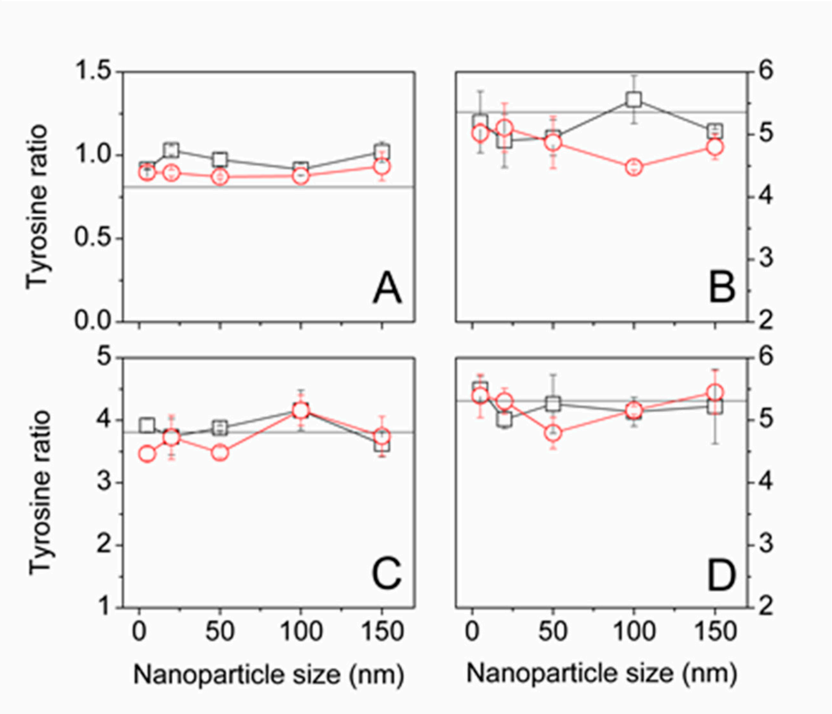

Figure S9. FTIR-ATR changes in tyrosine ratio for Mulberry (A), Eri (B), Muga (C), and Tasar (D) silks. Black squares (□) are $scCO_2$ samples, red circles (O) are control samples. The horizontal line is the tyrosine ratio in native silk. We found that for $scCO_2$ treatment alone, the tyrosine ratio was not significantly different from the native silks.

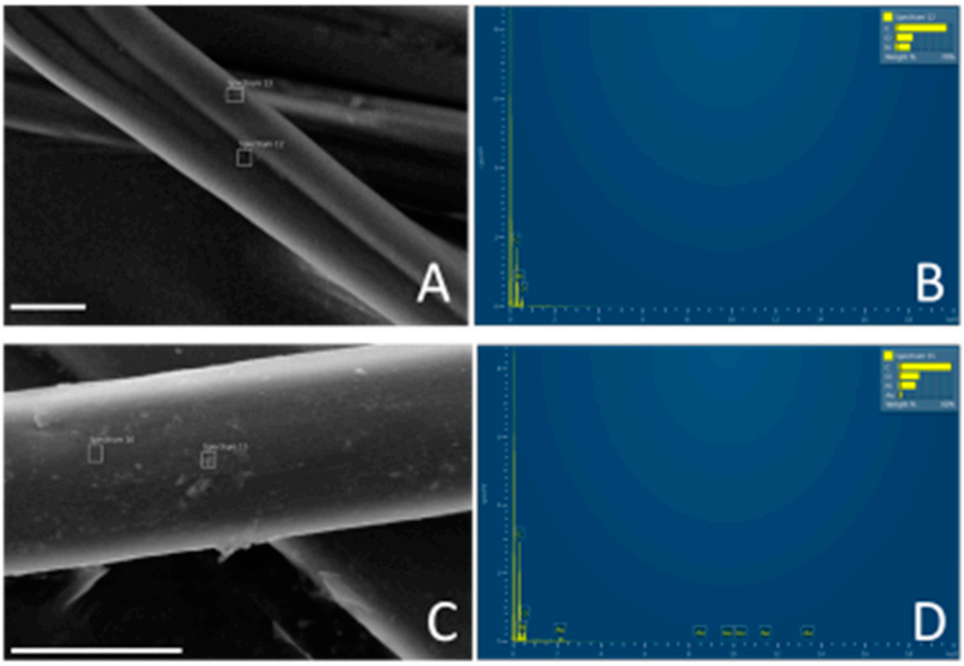

Figure S10. SEM images of native (**A**, scale bar 5μ) and impregnated (**C**, scale bar 10μ) silk fibers. The white squares represent the areas where the EDX spectra were collected. EDX spectra of native (**B**) and impregnated (**C**) silk fibers. The spectra showed only weak gold signals at 2.5, 7, 9.5, 10.5, 11.5, and 13.5 keV (**D**).

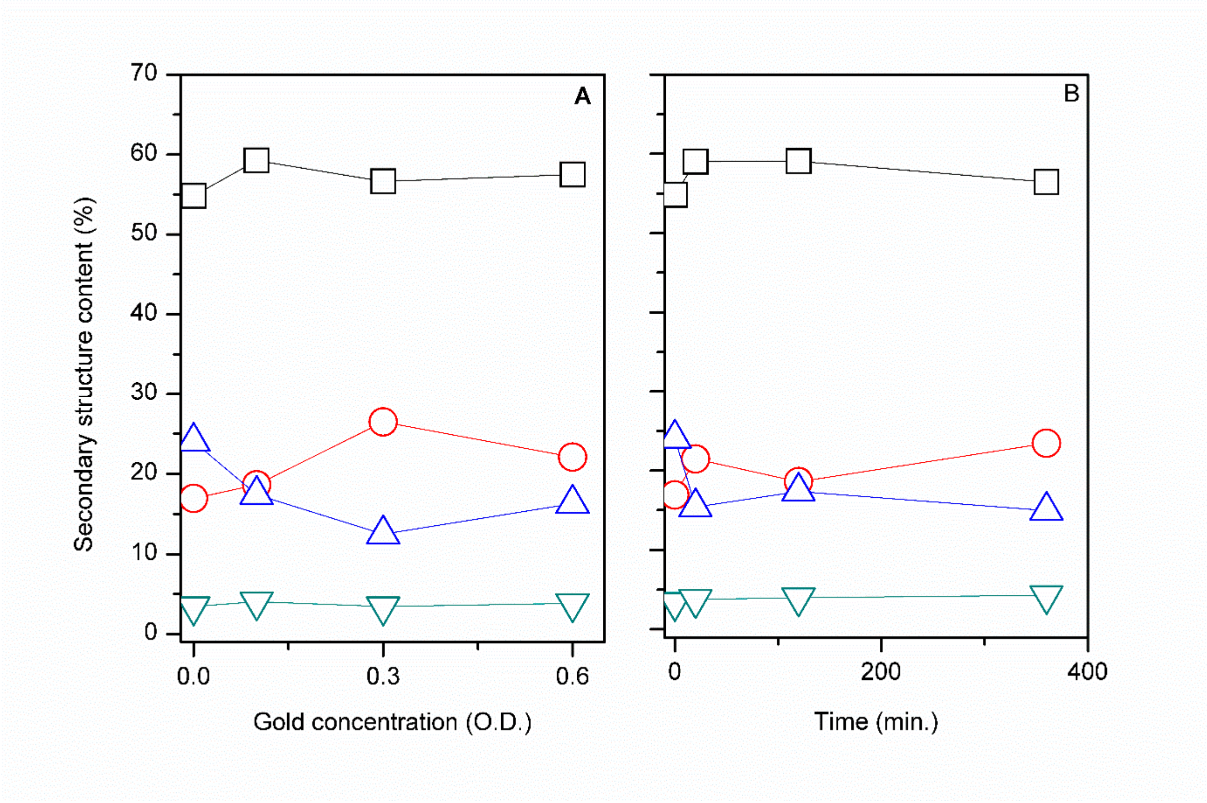

Figure S11. FTIR-ATR changes in secondary structure content as a function of Au NP concentration. Black squares (□) are inter β-sheets, red circles (O) are β-turns, blue triangles (Δ) are α-helices and random coils, inverted green triangles

(∇) are intra β-sheets.

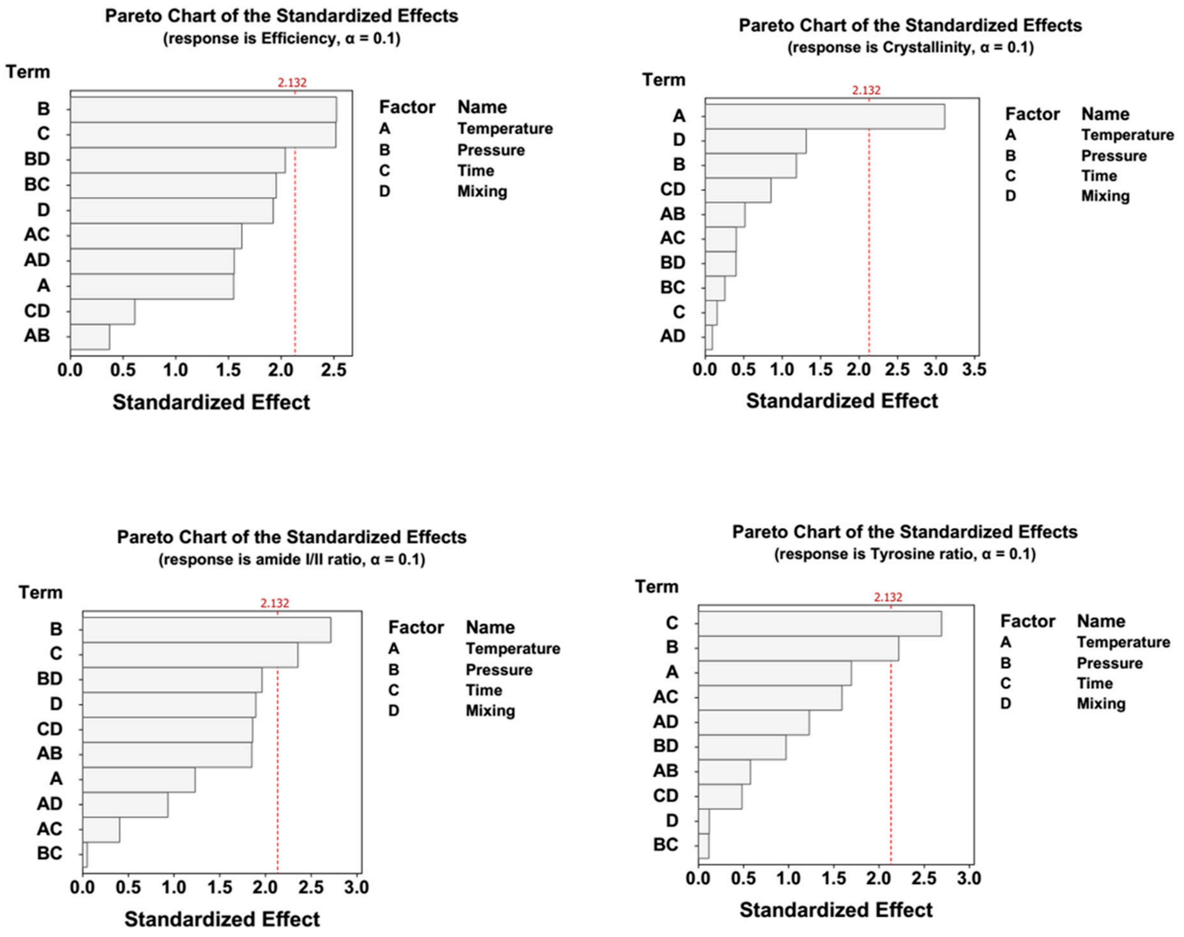

Figure S12. Pareto charts of the standardized effect from the full factorial analysis. The red dotted line is the standardized value above which a factor or combination of factors is considered significant (α level = 0.1).
